# A Novel Exopolysaccharide with Metal Adsorption Capacity Produced by a Marine Bacterium *Alteromonas* sp. JL2810

**DOI:** 10.3390/md15060175

**Published:** 2017-06-12

**Authors:** Zilian Zhang, Ruanhong Cai, Wenhui Zhang, Yingnan Fu, Nianzhi Jiao

**Affiliations:** State Key Laboratory of Marine Environmental Science, Institute of Marine Microbes and Ecospheres, Xiamen University, Xiamen 361102, China; crh1987@163.com (R.C.); qiaobamm@163.com (W.Z.); fuyingnan@xmu.edu.cn (Y.F.)

**Keywords:** exopolysaccharide, marine bacteria, *Alteromonas*, metal adsorption

## Abstract

Most marine bacteria can produce exopolysaccharides (EPS). However, very few structures of EPS produced by marine bacteria have been determined. The characterization of EPS structure is important for the elucidation of their biological functions and ecological roles. In this study, the structure of EPS produced by a marine bacterium, *Alteromonas* sp. JL2810, was characterized, and the biosorption of the EPS for heavy metals Cu^2+^, Ni^2+^, and Cr^6+^ was also investigated. Nuclear magnetic resonance (NMR) analysis indicated that the JL2810 EPS have a novel structure consisting of the repeating unit of [-3)-α-Rha*p*-(1→3)-α-Man*p*-(1→4)-α-3OAc-GalA*p*-(1→]. The biosorption of the EPS for heavy metals was affected by a medium pH; the maximum biosorption capacities for Cu^2+^ and Ni^2+^ were 140.8 ± 8.2 mg/g and 226.3 ± 3.3 mg/g at pH 5.0; however, for Cr^6+^ it was 215.2 ± 5.1 mg/g at pH 5.5. Infrared spectrometry analysis demonstrated that the groups of O-H, C=O, and C-O-C were the main function groups for the adsorption of JL2810 EPS with the heavy metals. The adsorption equilibrium of JL2810 EPS for Ni^2+^ was further analyzed, and the equilibrium data could be better represented by the Langmuir isotherm model. The novel EPS could be potentially used in industrial applications as a novel bio-resource for the removal of heavy metals.

## 1. Introduction

Exopolysaccharides (EPS) are high-molecular weight polymers consisting mainly of carbohydrates. The composition and structure of EPS vary widely; they may be homo- or hetero-polysaccharides and may contain a number of different organic and inorganic substituents such as sulfate, phosphate, acetic acid, and acetylate [1,2]. Most marine bacteria can produce EPS, which occur in two forms: capsular polysaccharides in which the polymers are covalently bound to the cell surface; and slime polysaccharides that either remain attached (loosely bound) to the cell surface or are released into the environment [3,4]. EPS produced by marine bacteria are thought to play a role in the protection against the marine environment characterized by extreme physicochemical conditions such as low or high temperature, high pressure, low nutrient concentration, high salinity, and heavy metal presence [2,5,6]. EPS also present a wide range of industrial applications such as in emulsification, thickening, gel formation, anticancer treatment, and metal biosorption [4,7,8]. For example, EPS from *Zoogloea* sp. possess excellent flocculating activity [9]; EPS from *Halolactibacillus miurensis* show high antioxidant activity [10]; over-sulfated EPS produced by *Alteromonas infernus* can inhibit the invasiveness of osteosarcoma cells [11]; and EPS from strain HYD657, *A. macleodii* subsp. fijiensis biovar deepsane, have been developed for cosmetic applications (patent PCT 94907582-4) [12,13]. However, until now very few EPS structures produced by marine bacteria have been determined [2], and the characterization of EPS structures is important for the elucidation of their biological functions and ecological roles.

Many microbial EPS show biosorption activity for metal ions. Currently, pollution from heavy metals has aroused intense concern due to the large amount released into the environment and the high risks to both the environment and human health [14,15]. For example, high levels of Ni^2+^ discharged from nickel smelting, acid mine drainage, and the electroplating industries affect the lungs and kidneys and generate several gastrointestinal and skin problems in human health [16]. Chromium (Cr^6+^) released from industries such as dyeing, leather tanning, and electroplating poses serious health risks from skin irritation to lung carcinoma [17]. As an important engineering material with wide industrial applications, excessive copper (Cu^2+^) in the marine system has been found to damage marine life. In humans, copper toxicity leads to a neurotoxic condition known commonly as “Wilson’s disease,” which occurs as a result of the deposition of copper in the lenticular nucleus of the brain [18]. Thus, the efficient removal of Ni^2+^, Cr^6+^, and Cu^2+^ from industrial wastewater is essential. Several methods have been developed to remove heavy metal ions from aqueous solutions such as ion-exchange, reverse osmosis, filtration, precipitation, coagulation, and biosorption [19]. Among these methods, biosorption is regarded as one of the most promising technologies due to its advantages of low cost, short operation time, and the reusability of biomaterials [20]. The adsorption of various heavy metals by EPS produced by bacteria such as *Bacillus firmus* [21], *Arthrobacter* ps-5 [22], cyanobacteria of *Synechocystis* sp. BAS0671 [23], *Anabaena spiroides* [24], and *Lyngbya putealis* [25] have been reported. Although many known EPS have shown adsorption for heavy metals, further investigation for new microbial EPS is promising. There is growing interest in isolating bacterial EPS with novel functions from the ocean; however, only a small number of these bacteria can be grown in culture and most of the marine bacterial world remains unexplored [26]. 

*Alteromonas* is a common marine heterotrophic γ-proteobacterium widely distributed in the ocean [27]. The production of EPS by several strains of *Alteromonas* has been characterized, and most strains were isolated from deep-sea hydrothermal vents [12,28,29,30,31]. We have previously isolated EPS-producing bacteria from surface seawater collected from the South China Sea. Among the isolates, bacterium *Alteromonas* sp. JL2810 produced the highest level of EPS [32], and the molecular weight and composition of JL2810 EPS were characterized [32]. In this study, the structure of JL2810 EPS was determined and the biosorption of the EPS for heavy metals of Cu^2+^, Ni^2+^, and Cr^6+^ was investigated. 

## 2. Results and Discussion

### 2.1. Structure Analysis of JL2810 EPS

The EPS produced by JL2810 had a high molecular weight (>1.67 × 10^5^) and contained large amounts of galacturonic acid (GalA) [32]. In this study, the structure of the purified EPS was determined by nuclear magnetic resonance (NMR) analysis. As substitution with *O*-acetyl groups increases the complexity of NMR spectra, removal of the acetyl groups is desirable. In this study, the removal of the *O*-acetyl groups was undertaken by treatment with 12.5% ammonium hydroxide solution overnight at room temperature. Comparison of the 2D NMR spectrum before and after the *O*-deacetylation allowed for the assignment of the *O*-acetyl location in the native material. Spectra were acquired at 70 °C, which provided better resolution and lowered the viscosity of the sample. 

**Figure 1:**
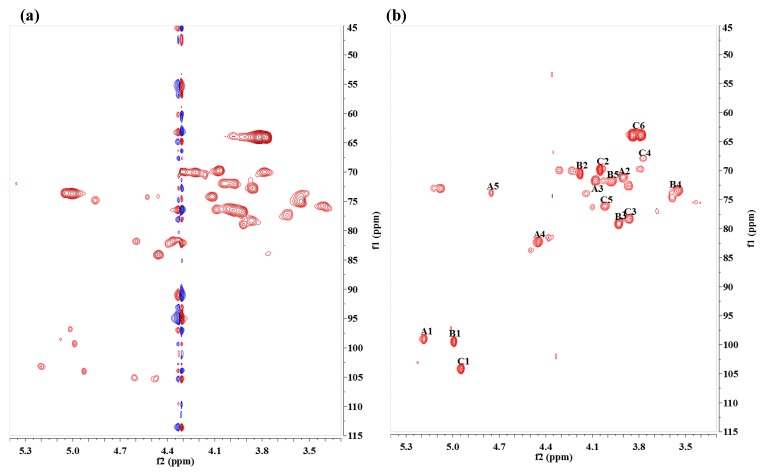
2D gHSQC spectrum of JL2810 EPS. (**a**) Native exopolysaccharides (EPS); and (**b**) *O*-deacetylated EPS.

The gHSQC spectrum showed three anomeric signals (A-H1, B-H1, C-H1) in the anomeric region (δ_H_ 4.4–5.5 ppm, δ_C_ 90–120 ppm) (Figure 1). Most signals were assigned after the interpretation of the gCOSY, TOCSY, ROESY, and gHMBC spectra (Figures S1–S4). 

Homonuclear COSY and TOCSY spectra were applied for the identification of individual monosaccharide residues. The gCOSY spectrum enabled the unambiguous assignment of all H2 protons from the H1/H2 cross-peaks (Figure S1). In the TOCSY experiment, each anomeric proton signal exhibited connectivity to all other signals for protons in the same ring (Figure S2). The intensity of connectivity depends on the magnitude of all the homonuclear coupling constants. For residue A, the weak H1/H4 cross-peak (δ_H1/H4_ 5.18/4.44 ppm) was observed due to the small coupling constant. The magnetization transferred from the anomeric proton was inefficient due to the equatorial H4; therefore, long-range connectivity between H1 and H2 through H4 was observed, which supported the assignment of GalA. In the same manner, residues B and C were assigned as rhamnose (Rha) and mannose (Man). Based on the chemical shifts of the anomeric signals, the three residues were assigned as α-GalAp, α-Rhap, and α-Manp.

The ROESY (Figure S3) and gHMBC (Figure S4) spectra provided information regarding the glycosidic linkage. The correlation signals at 5.18/3.93 (A-H1/B-H3), 4.99/3.87 (B-H1/C-H3), and 4.95/4.44 (C-H1/A-H4) in the ROESY spectrum revealed the presence of a [-3)-α-Rhap-(1→3)-α-Manp-(1→4)-α-GalAp-(1→] repeating unit. The appearance of cross-peaks in the gHMBC spectrum also confirmed the glycosidic linkage results.

Comparison of the NMR spectra before and after *O*-deacetylation showed that chemical shifts for H^1^ in the 4-α-GalAp residue were upfield and other protons in the ring were downfield, indicating that the *O*-acetyl group was attached at the 4-α-GalAp residue. Signals of H^3^ with higher ∆δ values when compared to other protons were observed, indicating that the *O*-acetyl group occurred at the 3-position. Chemical shifts are shown in Table 1.

**Figure 2:**
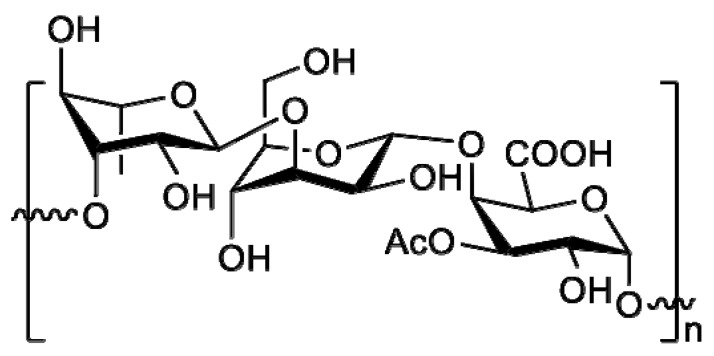
Chemical structure of the repeating unit of JL2810 EPS.

The polysaccharide was found to consist of a repeating unit of [-3)-α-Rhap-(1→3)-α-Manp-(1→4)-α-3OAc-GalAp-(1→] (Figure 2). 

Currently, only limited structural data for EPS produced by strains of *Alteromonas* are available, and nearly all of the strains were isolated from a deep-sea hydrothermal vent [13,28,33,34]. The EPS produced by the *Alteromonas* strains showed diverse composition and structure. For example, the EPS produced by strain ST716, *A. macleodii* subsp. *fijiensis*, were found to have a branched hexasaccharide repeating unit containing Glc, Gal, Man, GlcA, and GalA [34]. The repeating unit of EPS produced by the strain GY785 (belonging to the mesophilic species *A. infernus*) was a nonasaccharide with four types of monosaccharides: Glc, Gal, GalA, and GlcA [33]. In this study, we first determined the structure of the EPS produced by the *Alteromonas* strain JL2810 isolated from a normal marine environment. The structure of the EPS produced by JL2810 differed from that of EPS produced by other marine bacteria [35] and *Alteromonas* strains isolated from the deep-sea hydrothermal vent [13,28,33,34]. The repeating unit of EPS from JL2810 was a trisaccharide without branching and contained three types of monosaccharides (GalA, Man, and Rha). Residues of Gal and GlcA were identified in the EPS produced by deep-sea *Alteromonas* strains, but not in the EPS of JL2810. In contrast, the Rha residue was specifically identified in the EPS produced by JL2810. Only α-linkages were present in the EPS of JL2810; however, both α-and β-linkages were found in the EPS from deep-sea *Alteromonas* strains [13,28,33,34]. The EPS produced by JL2810 also differed from that produced by the moderately halophilic strain *A. hispanica* F23^T^ isolated from inland hypersaline habitats in Spain. The EPS form *A. hispanica* F23^T^ contained four types of monosaccharides (Glc, Man, Rha, and xylose) without uronic acid, and its structure has yet to be determined [36]. These results indicate that the EPS produced by JL2810 have a novel structure that differs from known EPS produced by other *Alteromonas* strains. 

As far as the microbial biodiversity is concerned, bacterial EPS exhibit a wide range of chemical structures [2]. Even closely related bacteria from different subspecies of *A. macleodii* produced very different EPS under the same growth conditions [12]. Based on the phylogenetic analysis results, the strain JL2810 was most closely related to *A. marina* SW-47^T^ [37]. Thus, the novel EPS produced by JL2810 may represent the characteristics of EPS produced by a species of *A. marina*. However, no EPS produced by *A. marina* has been reported and whether JL2810 belongs to the species of *A. marina* requires further analysis. Additional EPS produced by strains of *A. marina* should be further characterized.

### 2.2. Adsorption for Metal Ions

#### 2.2.1. Effect of pH 

**Figure 3:**
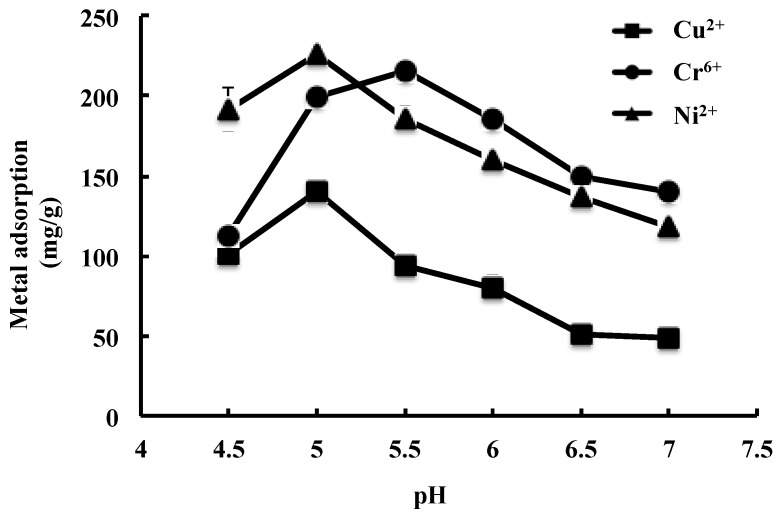
Effect of pH on the adsorption of JL2810 EPS for metals.

JL2810 EPS showed the adsorption capacity for Cu^2+^, Ni^2+^, and Cr^6+^ (Figure 3). The adsorption capacity for the metals was affected by pH. For Cu^2+^ and Ni^2+^, the maximum adsorptions were 140.8 ± 8.2 mg/g and 226.3 ± 3.3 mg/g at pH 5.0, but for Cr^6+^, this value was 215.2 ± 5.1 mg/g at pH 5.5. Similar trends were also observed by Ye [22] and Zhou [38]. The medium pH affects the solubility of metals and the ionization state of the functional groups of EPS. At low pH, the adsorption of heavy metals was possibly inhibited due to the competition between hydrogen and metals on the sorption sites. With an increase in pH, the negative charge density on the EPS surface may increase due to the deprotonation of the metal binding sites, and thus increase metal adsorption [39], which corresponded to the maximum adsorption capacity obtained at pH 5.0 for Ni^2+^ and Cu^2+^, and at pH 5.5 for Cr^6+^, respectively (Figure 3). The medium pH may also affect the ionization state of the function group of the EPS. It was indicated that carboxylic and phosphoric groups played a major role in the complexation of metal at pH 7.0, and the ability of EPS to complex heavy metals increased with the EPS phosphoric content [14]. However, based on the composition and structure analyses, no phosphoric group was detected in JL2810 EPS, which may be related to the relatively low adsorption capacity observed at pH 7.0. 

#### 2.2.2. FT-IR Analysis

**Figure 4:**
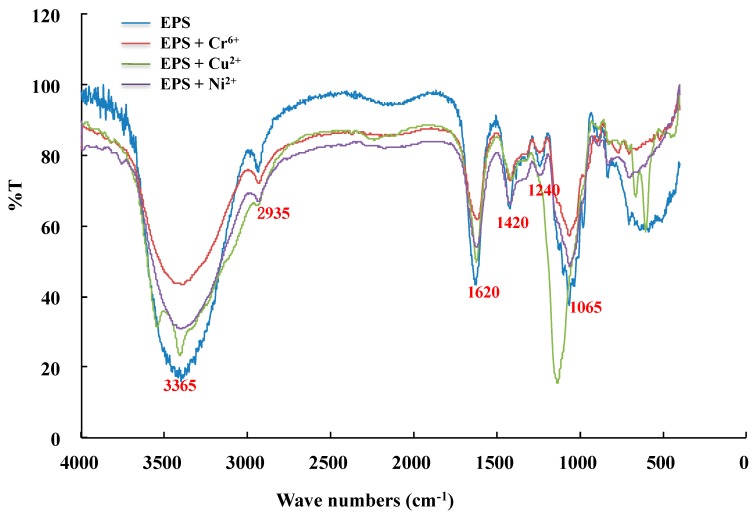
FT-IR spectra of JL2810 EPS before and after loading of Cu^2+^, Ni^2+^, and Cr^6+^.

The adsorption of heavy metals by EPS is energy independent, and can be caused by interaction between metal cations and functional groups of EPS. In this study, the functional group of JL2810 EPS for adsorption with the three heavy metals was analyzed by fourier transform infrared spectroscopy (FT-IR). As shown in the Figure 4, there were several distinct and characteristic sharp stretching frequencies of chemical groups contained in JL2810 EPS. The peak assignments of the EPS were as follows: 3400–3200 cm^−1^ was related to the stretch vibration of the O-H or hydrogen bond existing in all polymers; the signals at 3000–2800 cm^−1^ were related to the stretch vibration of the C-H bond; the signals at 1850–1600 cm^−1^ were due to the stretch vibration of the C=O bond; the signals at 1400–1370 cm^−1^ were attributed to the vibration of the C-O bond; the signals at 1240 cm^−1^ were due to the deformation vibration of the C=O bond; and the signals at 900–1150 cm^−1^ were for the C-O-C bond [14].

In comparing JL2810 EPS and metal-laden EPS, it was observed that there were shifts in wave numbers of dominant peaks at 3365 cm^−1^, 1620 cm^−1^, and 1065 cm^−1^, which represented the function groups of O-H, C=O, and C-O-C, respectively (Figure 4). These shifts in wave number corresponded to the metal binding process taking place on the surface of the adsorption. This is because the O of the polysaccharide structure complexes with metal in adsorption process to reduce the electron cloud density of functional groups containing oxygen and to change their vibration frequency and vibration intensity [14]. Based on the FT-IR analysis results, the groups of O-H, C=O, and C-O-C in JL2810 EPS played an important role in the adsorption for the heavy metals. 

We noticed that among the three metal-laden EPS samples, significant shifts were observed after the loading of Cu^2+^ (Figure 4). It was indicated that the Cu^2+^ easily bound the carboxyl groups of EPS [15]. These results suggested that the biosorption of EPS with heavy metals is selective, which depends on the structure and functional groups of the adsorbent as well as the state, size, and bond energy of metal ions [22]. 

The adsorption of heavy metals by EPS can be caused by the interaction between metals and the negative charge of the acidic function groups of EPS [38]. It was indicated that JL2810 EPS contained a large amount of GalA (42%) [32]. Similarly, it was indicated that the EPS produced by *Alteromonas* isolated from deep-sea hydrothermal vents also contained relatively large amount of acidic sugars such as GlcA and GalA [13,28,33,34]. The EPS that showed high metal adsorption capacity usually contained relatively large amounts of uronic acid. For example, the EPS from *Bacillus firmus* contained 38% uronic acid, and showed very high metal adsorption capacities (722–1103 mg/g) for Pb^2+^, Cu^2+^, and Zn^2+^ [21]. For cyanobacterium *Synechocystis* sp. BASO671, EPS production was increased by the metal ions of Cr^6+^ and Cd^2+^, and the composition of uronic acid in the cyanobacterial EPS was increased by Cr^6+^ and Cd^2+^ treatment [23]. In *Pseudomonas syringae*, Cu^2+^ induced the production of EPS (alginate) that contained high levels of uronic acid [40]. In contrast, the EPS from *A. hispanica* F23^T^ did not contain uronic acid, and their metal binding capacity was very low [36]. These results suggest that uronic acid GalA with the acetyl group in JL2810 EPS may play important roles in the adsorption of metals. 

#### 2.2.3. Adsorption Isotherms

**Figure 5:**
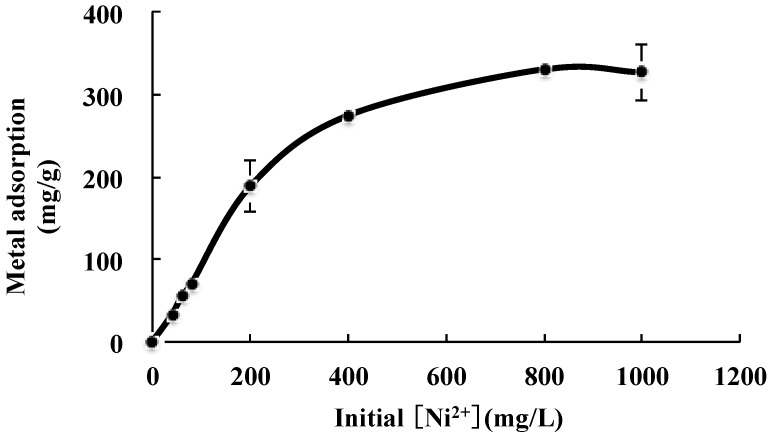
Effect of initial Ni^2+^ concentration on the adsorption with JL2810 EPS.

The initial metal concentration plays an important role in the process of biosorption. As shown in Figure 5, the adsorption capacity increased from 33.5 to 275 mg/g of EPS as the initial Ni^2+^ concentration increased from 40 mg/L to 400 mg/L. However, Ni^2+^ concentration above 400 mg/L did not increase adsorption significantly and remained almost constant (around 330 mg/L), indicating saturation of all the binding sites on the sorbent surface beyond a particular concentration (Figure 5). 

**Figure 6:**
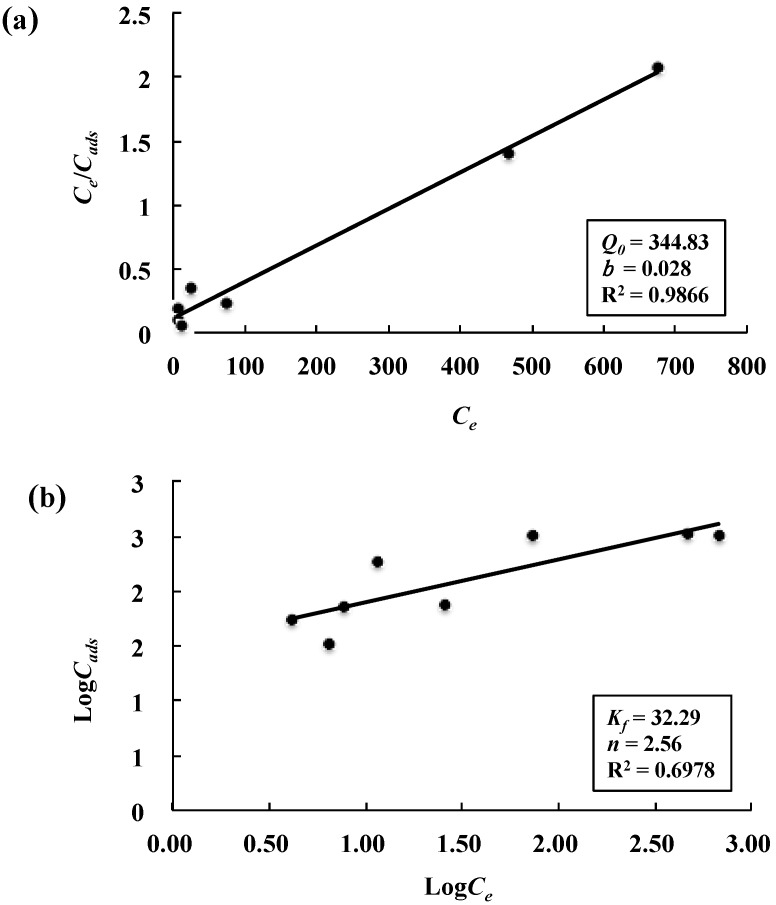
Linearized Langmuir and Freundlich adsorption isotherms for Ni^2+^ by the EPS: (**a**) Langmuir isotherm; and (**b**) Freundlich isotherm. The *C_ads_* is the amount of metal adsorbed per unit of dry weight (mg/g); *C_e_* is the metal concentration remaining in solution at equilibrium (mg/L).

The distribution of metal ions between the liquid phase and biosorbent in the equilibrium adsorption process can generally be described by several isotherms based on a set of assumptions related to the heterogeneity/homogeneity of adsorbents and the type of coverage [25]. Among these models, Langmuir and Freundlich models are those most frequently employed and were thus used in this study to describe the adsorption equilibrium of Ni^2+^ on JL2810 EPS. The Langmuir model is based on the assumption of monolayer adsorption onto a solid surface with a definite number of identical sites, while the Freundlich equation is based on the biosorption of an heterogeneous surface [24,25]. The linearized Langmuir and Freundlich adsorption isotherms along with the corresponding parameters evaluated from the isotherms are shown in Figure 6a,b, respectively. The adsorption capacity calculated from Langmuir equation (*Q*_0_ = 344.83 mg/g) is higher than that from Freundlich equation (*K_f_* = 32.29 mg/g), and closest to the maximum adsorption data observed (330 mg/g) (Figure 5). This fact indicated that the Langmuir isotherm model with a higher correlation coefficient (R^2^ = 0.9866) could better represent the equilibrium data than the Freundlich isotherm model (R^2^ = 0.6978). Similar results were reported by other studies, indicating that the biosorption of Cr^6+^ and Cu^2+^ to bacterial EPS could be better fitted by the Langmuir model than the Freundlich model [25,38]. Whether the Langmuir isotherm model is also suitable for describing the adsorption of the Cr^6+^ and Cu^2+^ on JL2810 EPS needs to be further studied. For the metal Ni^2+^, a dimensionless constant called separation factor (*R_L_*) was also calculated to test the favorability of adsorption, and the values of *R_L_* for the present biosorbent were in the range of 0.2–0.9, confirming the favorable adsorption of Ni^2+^ by JL2810 EPS. 

In the Langmuir model, the value of *Q*_0_ represents the maximum adsorption capacity and the value of *b* is related to the affinity of the binding sites. We noticed that both the values of *Q*_0_ and *b* were comparable with that of gelation for Pb^2+^, Cu^2+^, and Cd^2+^ with alginate (*Q*_0_: 167–435 mg/g; *b*: 0.011–0.046 L/mg), a highly promising material for the removal of heavy metals from wastewater [41]. The adsorption capacities of JL2810 EPS for Cu^2+^, Ni^2+^, and Cr^6+^ were also comparable to that of EPS produced by bacterial strains originating from deep-sea hydrothermal vents (77–316 mg/g for zinc, cadmium and lead) [42], but 3–5 times higher than that of EPS (40–48 mg/g for Cd^2+^ and Cu^2+^) produced by the marine bacterium *Wanggia profunda* SM-87 [38]. The high metal adsorption capacity of JL2810 EPS suggested that the EPS have a potential use in industrial applications as a novel bio-resource for the removal of heavy metals from polluted environments.

Furthermore, it was indicated that at the pH of ambient seawater, anionically charged EPS could remove more than 99% of heavy metals such as zinc and silver [43]. The binding of dissolved ions by EPS is considered essential in the vertical transport of metal ions in the ocean [44]. Since *Alteromonas* is widely distributed in the ocean [27] and can produce large amounts of EPS [32], the acidic EPS produced by *Alteromonas* may be important for the metal cycle in the ocean. 

## 3. Materials and Methods 

### 3.1. Bacterial Cultivation

*Alteromonas* sp. JL2810 isolated from the surface seawater of the South China Sea [32] was used for EPS isolation. A basal medium (BM) with glucose (Glc) as a sole carbon source was used to culture JL2810 and EPS production. The BM contained 1.9% NaCl, 0.03% NH_4_Cl, 0.03% KCl, 0.04% K_2_HPO_4_, 0.05% MgSO_4_·7H_2_O, 0.003% CaCl_2_·7H_2_O, and 1% Glc, pH 7.6. The cultivation was performed using a 3.7 L KLF 2000 bioreactor (Bioengineering, Wald, Switzerland), as described previously in Reference [32]. 

### 3.2. EPS Isolation and Purification

After cultivation, bacterial cells were removed from the culture medium by centrifugation (8000× *g*, 20 min) and filtered through a 0.22-μm Millex-GF filter unit. Three volumes of cold ethanol (–20 °C) were added to one volume of supernatant and the mixture was incubated at 4 °C overnight. The precipitate was harvested by centrifugation (12,000× *g*, 30 min), washed with 75% ethanol three times, and then dissolved in water. Proteins were removed using the Sevag method [45]. After centrifugation, the EPS solution was dialyzed (3500 Da cutoff) against water for 24 h. Next, the EPS were re-precipitated with three volumes of ethanol at 4 °C overnight and then harvested by centrifugation as mentioned above. The EPS were freeze-dried and used as crude EPS. 

The crude EPS were dissolved in water at a concentration of 5 g/L and purified using anion-exchange chromatography with a DEAE-Sepharose Fast Flow column (1.6 × 30 cm) (GE Healthcare, Little Chalfont, UK). EPS were eluted with 20 mL of H_2_O at a flow rate of 0.5 mL/min followed by a NaCl gradient (30 mL) ranging 0–2 M at a flow rate of 1 mL/min. The process was carried out on an AKTA purifier FPLC system (Amersham Biosciences, Amersham, UK). Each elution fraction (2 mL) was collected, and the EPS content was assayed using the phenol/sulfuric acid method. Major EPS-containing fractions were combined. After dialysis against distilled water, the EPS were freeze-dried and used as purified EPS. 

### 3.3. EPS Structure Analysis

The glycosyl-linkages of the purified EPS were analyzed. Both native and *O*-deacetylated EPS were analyzed by nuclear magnetic resonance (NMR). The native sample was deuterium-exchanged twice by lyophilization in D_2_O (99.9% D, Aldrich, St. Louis, MO, USA), and dissolved in 0.5 mL D_2_O (99.96% D, Cambridge Isotope, Tewksbury, MA, USA). Next, 10 μL of 20% acetone in D_2_O was added and placed into a 5-mm NMR tube. An *O*-deacetylated sample was also prepared for NMR analysis to elucidate the structure. The sample was adjusted to pH 11.0 by adding 12.5% ammonium hydroxide solution and stored overnight at room temperature. Next, dialysis (2000 Da cutoff) was applied to remove the salt. The sample was deuterium-exchanged as described above. 

NMR spectra were acquired at 600 (^1^H) or 150 MHz (^13^C) on a Biofuel-600 instrument at 70 °C (Agilent Technologies, Santa Clara, CA, USA). One-dimensional (1D) proton and two-dimensional (2D) gradient correlation spectroscopy (gCOSY), total correlation spectroscopy (TOCSY), rotating-frame overhauser effect spectroscopy (ROESY), gradient heteronuclear single quantum correlation spectroscopy (gHSQC), and gradient heteronuclear multiple bond correlation (gHMBC) spectra were acquired. Chemical shifts were referenced relative to a standard 4,4-dimethyl-4-silapentane-1-sulfonic acid (DSS) (0.000 ppm) by setting the chemical shifts of acetone to 2.218 ppm in the proton dimension and 33.0 ppm in the carbon dimension. The 1D proton spectrum was signal-averaged from 64 scans. The 2D gCOSY, TOCSY, and NOESY spectra were acquired in 32 scans per increment for 96 increments, and the gHSQC and gHMBC spectra were acquired in 256 scans and 64 increments. Spectral width was 4500 Hz for the proton spectrum and 11,000 Hz for the carbon spectrum. Acquisition time was 2.5 s for the 1D proton spectrum and 150 ms for the 2D spectra. The mixing time for the TOCSY experiment was 80 ms.

### 3.4. Metal Adsorption Experiments

#### 3.4.1. Effect of pH on the Metal Adsorption 

The adsorption capacity of the EPS for heavy metals was evaluated as described by Salehizadeh and Shojaosadatiet with some modification [21]. Stock solutions of Cu^2+^, Cr^6+^, and Ni^2+^ (1 g/L) were prepared by dissolving specific quantities of CuSO_4_, K_2_Cr_2_O_7_, and NiCl_2_ in deionized water and working standards were obtained by further dilution. The adsorption experiment was carried out by adding 1.5 mL EPS solution (1 g/L) and 1.5 mL heavy metal solution (1 g/L) in a 15-mL centrifuge tube and then on a shaker at 200 rpm. After incubation for 15 min, three volumes of chilled ethanol were added and the sample was centrifuged at 10,000× *g* for 10 min. Next, residual Cu^2+^, Cr^6+^, and Ni^2+^ in the supernatant were determined using an inductively coupled plasma atomic emission spectrometer (Optima 7000 DV, Perkin Elmer, Waltham, MA, USA). To investigate the effect of pH on the adsorption, the pH of the metal solution was adjusted to 4.5–7.0 by HCl or NaOH. All experiments were carried out in triplicate, the experimental data were presented as mean values ± standard deviations. Treatment without EPS solution was used as a control.

The amount of a metal adsorbed onto JL2810 EPS at equilibrium, *Q* (mg/g), was calculated using the equation *Q* = (*C_i_* − *C_e_*)/C*_eps_*, where *C_i_* and *C_e_* are the initial and residual concentrations of metals, respectively, and *C_eps_* is the EPS concentration.

#### 3.4.2. Infrared Analysis 

The infrared (IR) spectrum of EPS was determined using a fourier transform infrared spectrophotometer Thermo Nicolet Avatar 370 (Thermo Nicolet, Madison, WI, USA). Samples of purified EPS and metal-laden EPS were prepared for FT-IR measurement. To prepare the metal-laden EPS, purified EPS were incubated with metals of Cu^2+^, Ni^2+^, and Cr^6+^ as described above. After incubation for 15 min, chilled ethanol was added to the solution, and then sample was centrifuged at 10,000× *g* for 10 min. The pellet was washed by 70% ethanol twice, and then freeze-dried. The freeze-dried sample was ground with KBr powder and pressed into pellets for FT-IR measurement in the frequency range of 4000–400 cm^−1^ [22].

#### 3.4.3. Analysis of Adsorption Isotherms 

Adsorption isotherms of JL2810 EPS with Ni^2+^ were analyzed. Briefly, JL2810 EPS were incubated with different Ni^2+^ concentrations ranging from 0 to 1000 mg/L (pH 5.0). Other processes were the same as described above. The adsorption of Ni^2+^ with the EPS was analyzed using two models; Langmuir and Freundlich [24,25]. 

For the Langmuir model, the equation can be represented as *C_ads_* = *Q*_0_*bC_e_*/(1 + *bC_e_*). *C_ads_* is the amount of metal adsorbed per unit of dry weight (mg/g); *C_e_* is the metal concentration remaining in solution at equilibrium (mg/L); *Q*_0_ (mg/g) and *b* (L/mg) are the Langmuir constants that can be determined from the linear plot of *C_e_*/*C_ads_* versus *C_e_*. *Q*_0_ represents the maximum adsorption capacity when the surface is fully covered with metal ions and the constant *b* is related to the affinity of the binding sites [25]. Furthermore, a dimensionless constant called the separation factor (*R_L_*) was also calculated to test the favorability of adsorption as per the equation of *R_L_* = 1/(1 + *bC_i_*), where *b* is the Langmuir isotherm constant and *C_i_* is the initial metal ion concentration (mg/L). This parameter explains the isotherm as follows: *R_L_* > 1, unfavorable; RL = 1, linear; 0 < *R_L_* < 1, favorable; *R_L_* = 0, irreversible [25].

For the Freundlich model, the equation can be represented as log*C_ads_* = log*K_f_* + n^−1^(log*C_e_*). *C_ads_* and *C_e_* are the same as described above. *K_f_* is the Freundlich constant indicating adsorbent capacity (mg/g) and *n* is Freundlich exponent known as adsorbent intensity. The constant *K_f_* and *n* can be determined from the linear plot of log*C_ads_* versus log*C_e_* [25].

## 4. Conclusions

In this study, the structure of JL2810 EPS was analyzed by NMR analysis. The data indicated that EPS have a novel structure consisting of a repeating unit of [-3)-α-Rha*p*-(1→3)-α-Man*p*-(1→4)-α-3OAc-GalA*p*-(1→]. The EPS showed high adsorption capacity for Cu^2+^, Ni^2+^, and Cr^6+^. The adsorption of the EPS for heavy metals was affected by a medium pH. The groups of O-H, C=O, and C-O-C were the main function groups for adsorption of JL2810 EPS with the heavy metals. The Langmuir and Freundlich models were employed to describe the biosorption equilibrium data for Ni^2+^, and it was found that the equilibrium data could be better represented by the Langmuir isotherm model. The high adsorption capacity suggested that JL2810 EPS has potential in industrial applications as a novel bio-resource for the removal of heavy metals.

## Figures and Tables

**Table 1:** Chemical shift of signals in ^1^H and ^13^C NMR spectra of JL2810 EPS.

EPS	Compoumd	Residue	Nuclear	Chemical Shift (ppm)						NOE
				1	2	3	4	5	6	*HMBC*
**Deacetylted**	**A**	**4-α-GalAp**	**^1^H**	5.18	3.9	4.08	4.44	4.75	-	B-H3
			**^13^C**	*99*	*71*	*71.3*	***82.4***	*73.7*	*176.2*	-
	**B**	**3-α-Rhap**	**^1^H**	4.99	4.19	3.93	3.56	3.98	1.27	C-H3
			**^13^C**	*99.3*	*70.4*	***79***	*73.4*	*71.7*	*19.6*	C*-C3*
	**C**	**3-α-Manp**	**^1^H**	4.95	4.05	3.87	3.77	4.02	3.83/3.79	A-H4
			**^13^C**	*104*	*69.8*	***78.2***	*67.7*	*75.9*	*63.8*	A*-C4*
**Native EPS**	**A’**	**3-OAc-4-α-GalAp**	**^1^H**	5.09	3.98	**5.07**	4.61	4.88	-	
			**^13^C**	*98.5*	*71.9*	*73.5*	***81.7***	*74.7*	*176.2*	

Carbon chemical shifts in italics; glycosylated, downfield carbon resonances in bold.

## Supplementary Materials

The following are available online at www.mdpi.com/1660-3397/15/6/175/s1, Figure S1: 2D gCOSY spectra of native and deacetyled JL2810 EPS, Figure S2: 2D TOCSY spectra of native and deacetyled JL2810 EPS, Figure S3: 2D ROESY spectra of native and deacetyled JL2810 EPS, Figure S4: 2D gHMBC spectrum of deacetyled JL2810 EPS.
