# Supplementary material for: A Novel Exopolysaccharide with Metal Adsorption Capacity Produced by a Marine Bacterium Alteromonas sp. JL2810
Source: Mar Drugs. 2017 Jun 12;15(6):175. doi: 10.3390/md15060175 (PMC5484125; doi:10.3390/md15060175)
Supplement: Supplementary file 1 [file marinedrugs-15-00175-s001.pdf]

Supplemental data

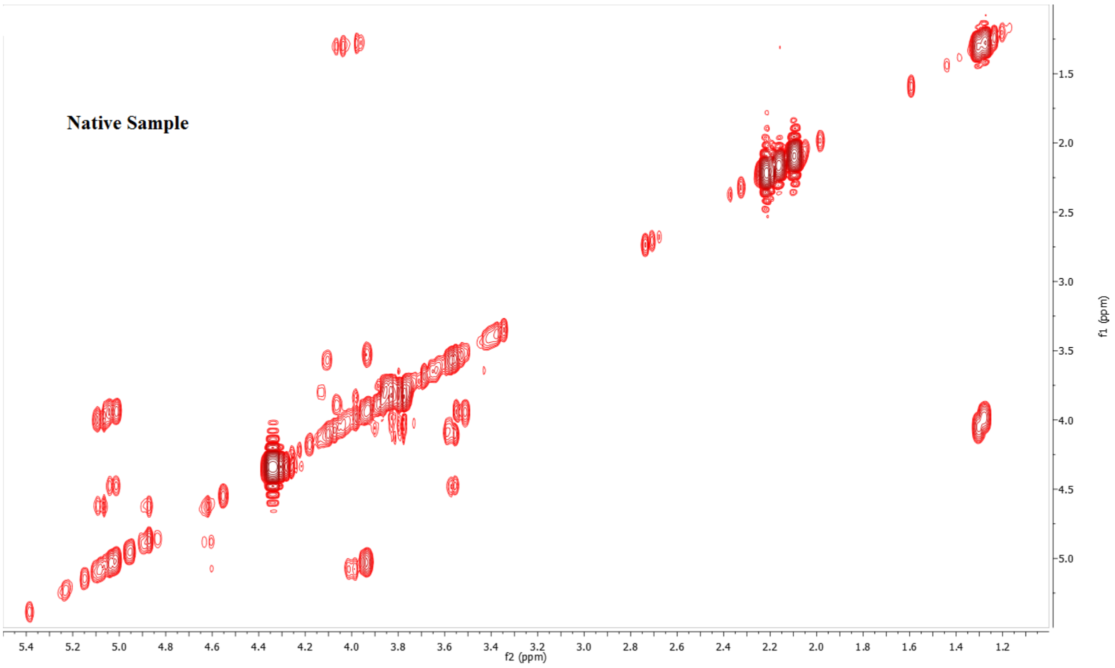

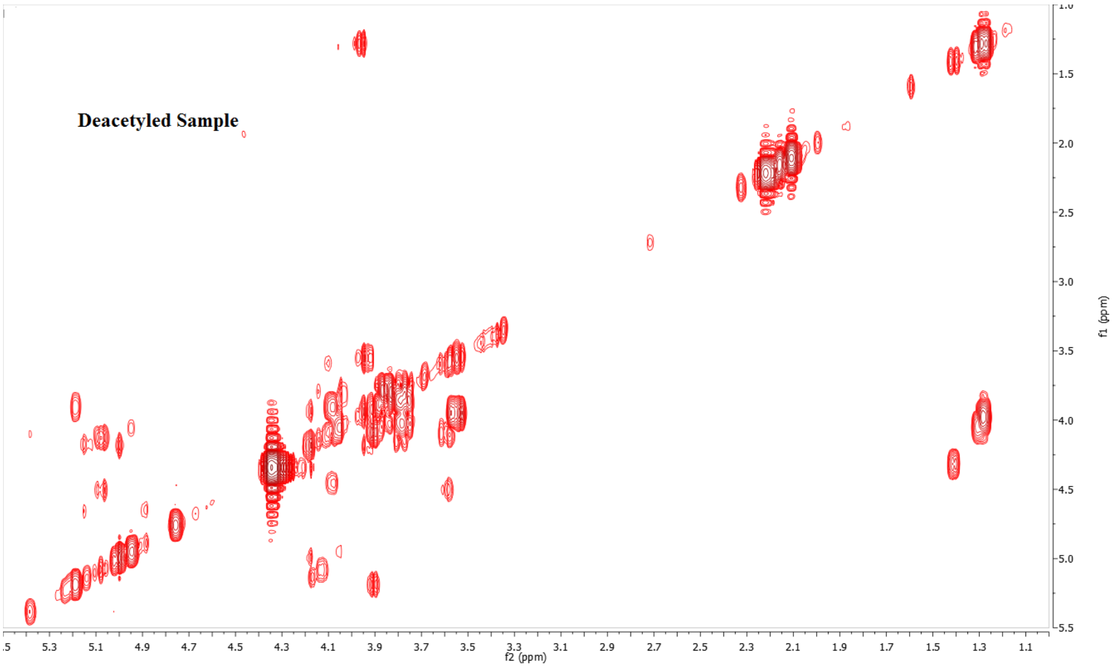

Figure S1. 2D gCOSY spectra of native and deacetyled JL2810 EPS.

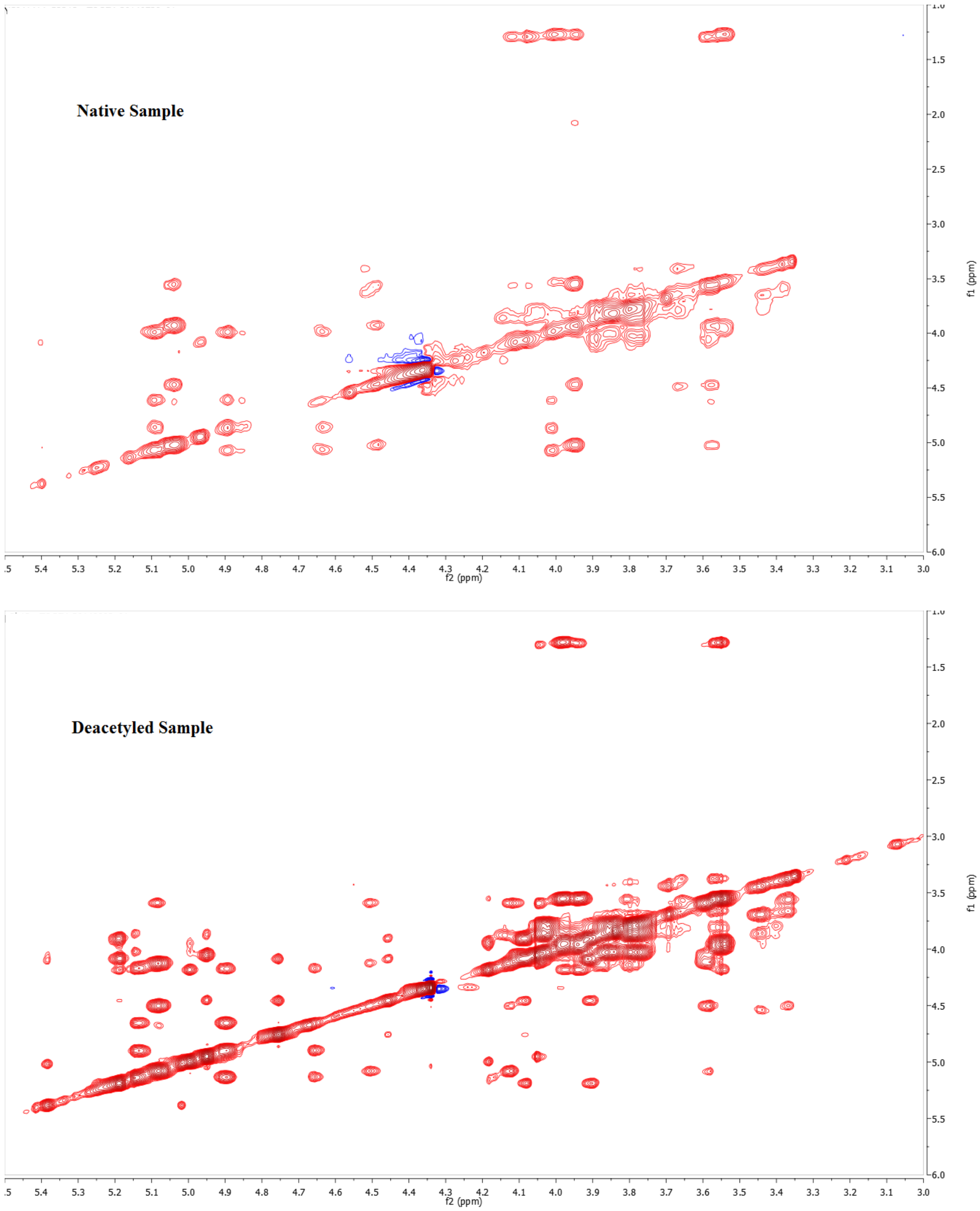

Figure S2. 2D TOCSY spectra of native and deacetyled JL2810 EPS.

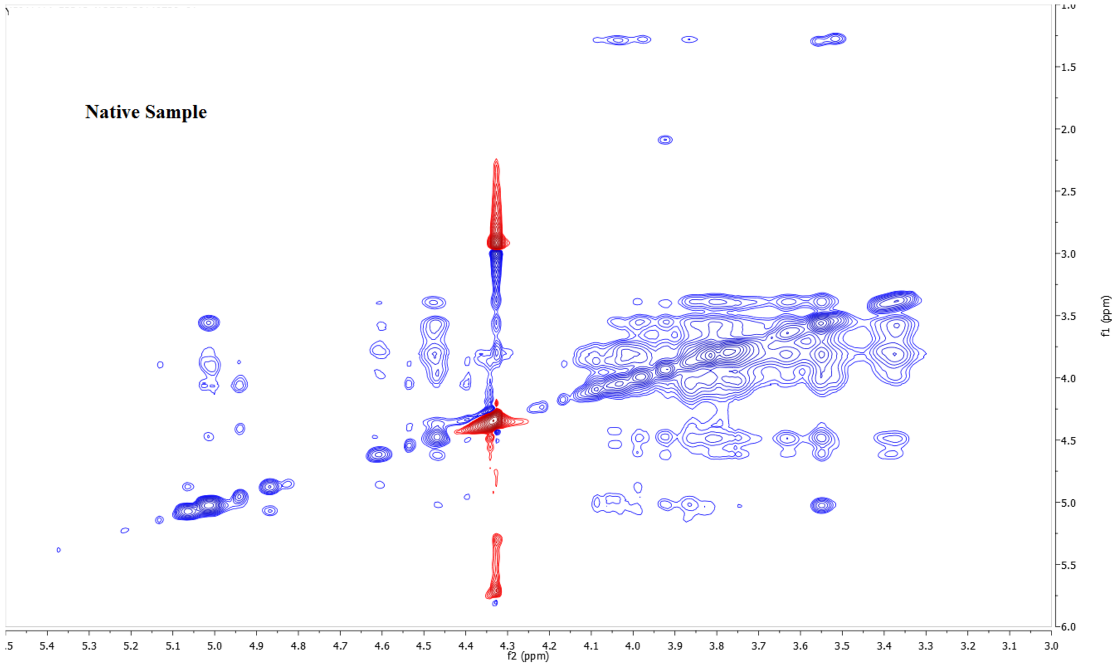

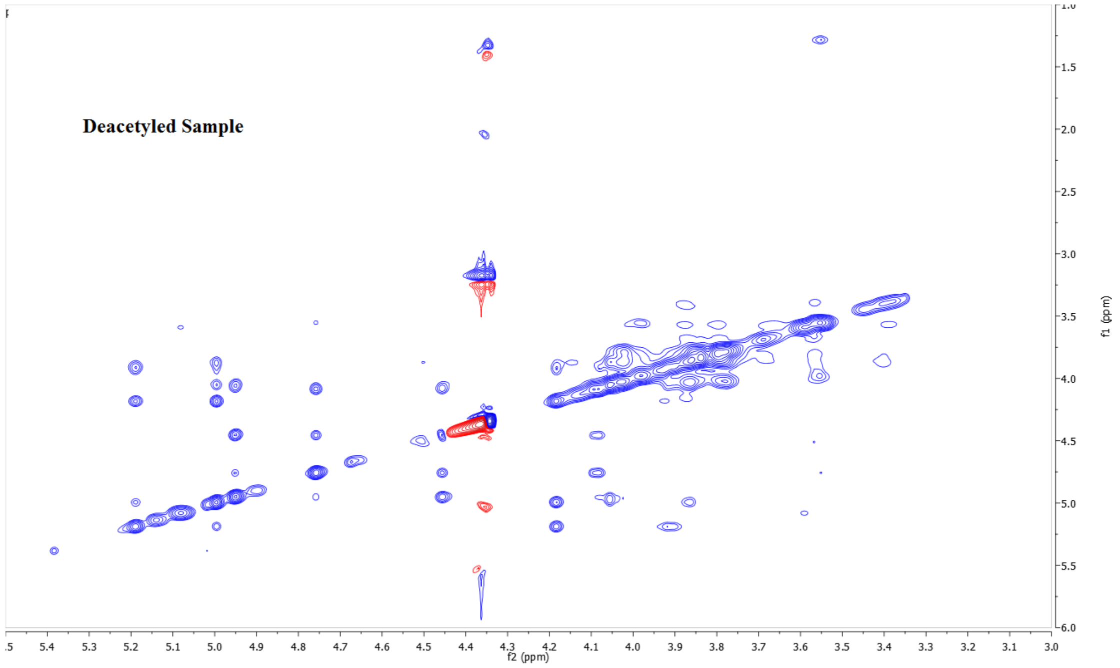

Figure S3. 2D ROESY spectra of native and deacetyled JL2810 EPS.

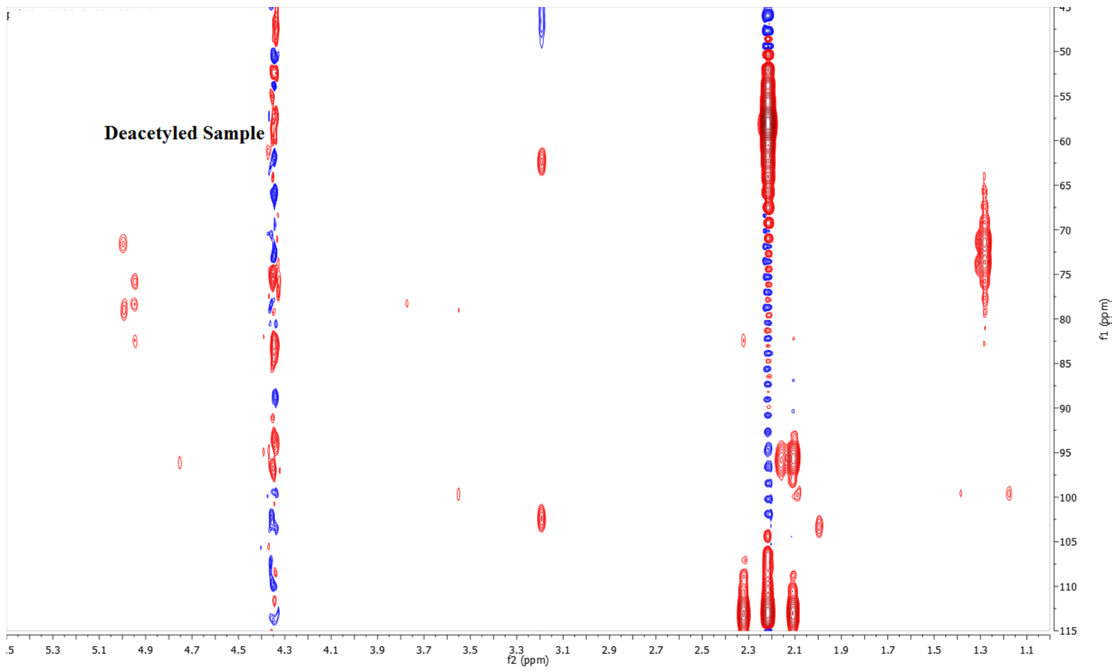

Figure S4. 2D gHMBC spectrum of deacetyled JL2810 EPS.
